# The protection of meloxicam against chronic aluminium overload-induced liver injury in rats

**DOI:** 10.18632/oncotarget.15588

**Published:** 2017-02-21

**Authors:** Yang Yang, Qin He, Hong Wang, Xinyue Hu, Ying Luo, Guojuan Liang, Shengnan Kuang, Shaoshan Mai, Jie Ma, Xiaoyan Tian, Qi Chen, Junqing Yang

**Affiliations:** ^1^ Department of Pharmacology, Chongqing Medical University, The Key Laboratory of Biochemistry and Molecular Pharmacology, Chongqing 400016, China; ^2^ Department of Hepatobiliary Surgery, 1st Affiliated Hospital, Chongqing Medical University, Chongqing 400016, China

**Keywords:** chronic aluminium load, liver function, inflammation, oxidative stress

## Abstract

The present study was designed to observe the protective effect and mechanisms of meloxicam on liver injury caused by chronic aluminium exposure in rats. The histopathology was detected by hematoxylin-eosin staining. The levels of prostaglandin E2, cyclic adenosine monophosphate and inflammatory cytokines were detected by enzyme linked immunosorbent assay. The expressions of cyclooxygenases-2, prostaglandin E2 receptors and protein kinase A were measured by western blotting and immunohistochemistry. Our experimental results showed that aluminium overload significantly damaged the liver. Aluminium also significantly increased the expressions of cyclooxygenases-2, prostaglandin E2, cyclic adenosine monophosphate, protein kinase A and the prostaglandin E2 receptors (EP_1,2,4_) and the levels of inflammation and oxidative stress, while significantly decreased the EP3 expression in liver. The administration of meloxicam significantly improved the impairment of liver. The contents of prostaglandin E2 and cyclic adenosine monophosphate were significantly decreased by administration of meloxicam. The administration of meloxicam also significantly decreased the expressions of cyclooxygenases-2 and protein kinase A and the levels of inflammation and oxidative stress, while significantly increased the EP_1,2,3,4_ expressions in rat liver. Our results suggested that the imbalance of cyclooxygenases-2 and downstream prostaglandin E2 signaling pathway is involved in the injury of chronic aluminium-overload rat liver. The protective mechanism of meloxicam on aluminium-overload liver injury is attributed to reconstruct the balance of cyclooxygenases-2 and downstream prostaglandin E2 signaling pathway.

## INTRODUCTION

Aluminium (Al) is widely used as a food and drug additive [1]. Therefore, humans inevitably come into frequent contact with Al. Excessive Al may cause damage to the body's tissues and organs *e.g*. renal, bone, lung, heart and central nervous system [2–3]. Previous study showed that the Al accumulation in the liver may be more obvious than brain and other organs [4]. In liver, Al is mainly accumulated in macrophages and lysosomes. Considering that Al in lysosomes can be eliminated by hepatocytes via bile excretion, many scholars believe that Al accumulation does not cause significant liver toxicity [5]. So, hepatotoxicity from long-term exposure to Al has not received sufficient attention. However, recent studies showed that Al administration (50 mg Al per rat, ip) caused significant accumulation of Al in rat hepatic cells, mild hyperplasia of bile duct and liver fatty degeneration [6]. Viezeliene also found that treatment with Al (ip) resulted in a four-fold increase in alanine aminotransferase (ALT) levels and a significant decrease in glutathione (GSH) activity in mice compared to normal controls [7]. Intragastric administration of AlCl_3_ (34 mg·kg^−1^ for 30 days) caused a significant increase in ALT, alkaline phosphatase(ALP), aspartate aminotransferase(AST) and lactic dehydrogenase(LDH) levels in rat serum and resulted in obvious damage to hepatic cells, including liver blood sinus expansion, central venous hyperemia, lipid accumulation and lymphocyte infiltration [8]. Furthermore, with a daily injection of 37.5 mg AlCl_3_·kg^−1^, degeneration became evident in the livers of rats (*e.g*., obvious karyopyknosis and cell loss) [9]. These results indicate that Al overload can also cause significant changes in liver morphology and function. However, the mechanism of damage to liver is still unclear.

Trivalent Al could react with water to produce bidentate superoxide coordination spheres [Al (O_2_) (H_2_O_4_)^2+^ and Al (H_2_O)_6_^3+^] that after complexation with O^2−^, generate Al superoxides [Al(O^2−^)](H_2_O_5_)]^2+^. Semi reduced AlO_2_ radicals deplete mitochondrial Fe and promote generation of H_2_O_2_, O_2_ and OH^−^. So, it is the Al^3+^-induced formation of oxygen radicals that accounts for the oxidative damage that leads to intrinsic apoptosis [10]. Al induced oxidative stress in tissues like brain, kidney and liver by decreasing intracellular GSH [11]. Various Al salt complexes could provoke macrophage responsibility which promoted an inflammatory cascade [12]. Our previous study also showed that chronic intragastric administration of Al gluconate (Al^3+^ 200 mg·kg^−1^ per day, 5 d a week for 20 weeks) causes obvious damage to rat liver and that metal ion imbalance-related oxidative stress may be involved in the mechanism of chronic liver injury caused by Al overload [13]. The relief of oxidative stress could alleviate liver injury induced by Al [14–16]. Those results indicated that the toxicity effect of Al is closely related with inflammation and oxidative stress.

Arachidonic acid (AA) is the precursor of a number of biologically active substances, including prostaglandins(PGs), leukotrienes(LTs), and *etc* which have important effects on immune and inflammatory systems. 5-lipoxygenase is a metabolic pathway of AA to produce LTs. Our previous research showed that 5-lipoxygenase inhibitors could significantly alleviate inflammation and oxidative stress in chronic Al-overload liver injury [17]. However, cyclooxygenase (COX) pathway is an another very important metabolic pathway of AA. COX includes the structural COX1 and the inducible COX2. The level of COX2 expression is low in normal situation, but it can significantly increase under a variety of pathological conditions [18]. The expression of COX2 of liver tissue was significantly induced by chronic hepatitis C virus and participated in the process of liver inflammation, necrosis, fibrosis, and the hepatocellular carcinoma development caused by the viral infection [19]. COX2 expression was also increased in some non-infectious liver injury such as carbon tetrachloride intoxication, alcoholic and non-alcoholic steatohepatitis [20–21]. Prostaglandin E2 (PGE2) is one of the most abundant and active of the PGs in COX2 downstream. Those results indicated that COX2-PGE2 pathway may participate in pathophysiological process of chronic liver damage and that COX2 may be considered as a novel therapeutic target for liver injury.

However, the relationship between COX2 expression and Al-overload liver damage, and the observation of protective effect of COX2 inhibitor on liver injury caused by chronic Al overload have never been reported. Therefore, the present study was designed to observe the change of COX2 and downstream PGE2 signaling pathway of rat liver and the protective effect of meloxicam, a selective COX2 inhibitor, on liver injury in chronic Al overload rat. The results of this present study will contribute to explore the mechanism of the hepatotoxicity for long-term exposure to Al and to seek new therapeutic strategy for chronic non-infectious liver damage.

## RESULTS

### Hepatic histopathology

**Figure 1:**
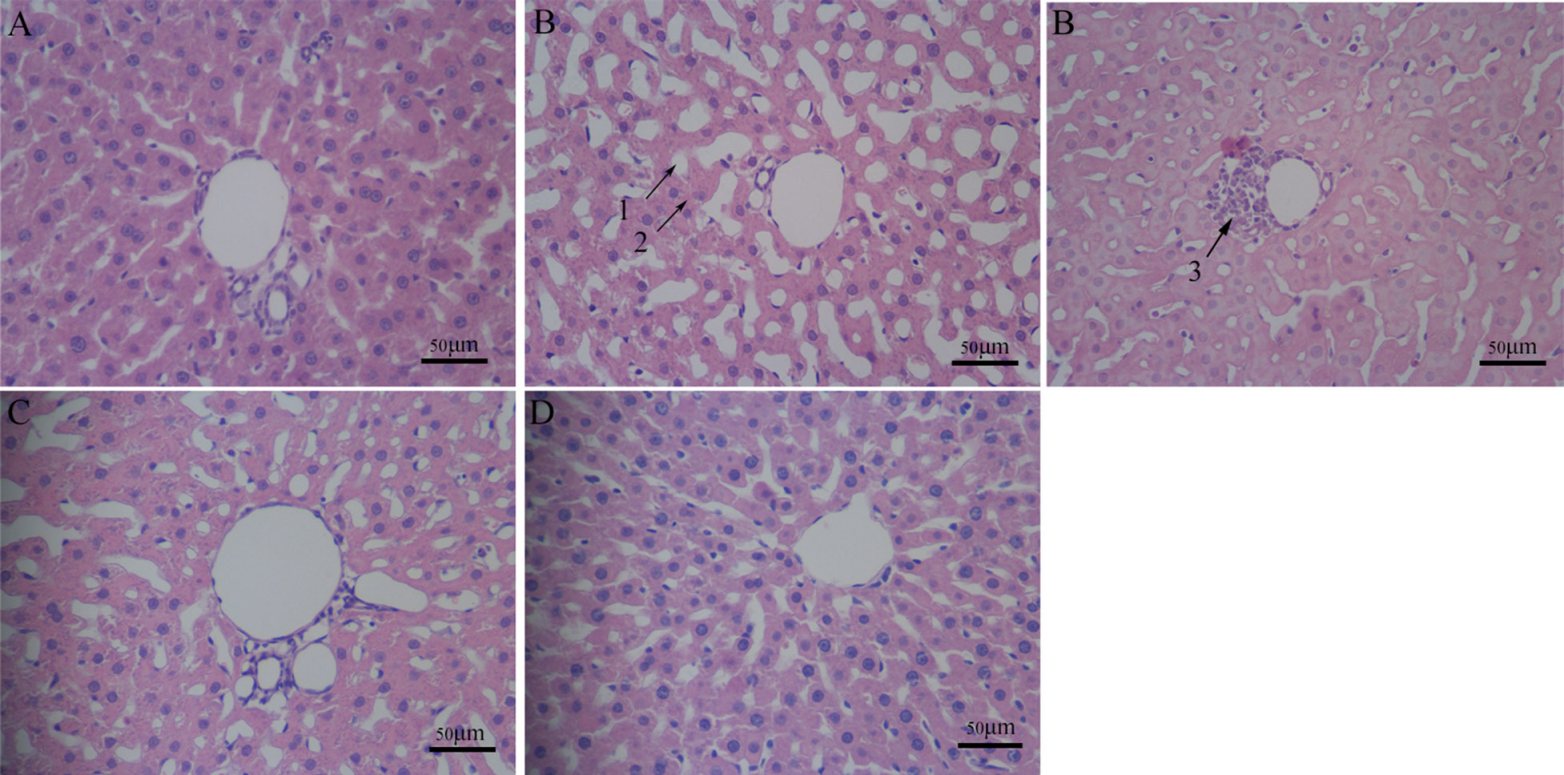
Pathomorphology in rat liver Hepatocyte pathomorphology in chronic Al overload rat livers (HE × 400, Scale bars = 50 μm). (**A**) Control group; regular histological features of liver of control group with well formed central vein, cord like arrangement of hepatocytes around the central vein. (**B**) Model group; regular histological features of liver have changed, section of Al exposed liver with several irregularities: 1. vacuolar degeneration, 2. granular degeneration, 3. spotty necrosis. (**C**) Al + Meloxicam 0.3 mg.kg^−1^ group; the administration of meloxicam significantly alleviated the hepatocyte injury of the vacuolar degeneration, spotty necrosis and granular degeneration. (**D**) Al + Meloxicam 1 mg.kg^−1^ group; the hepatocyte injury was alleviated and the hepatic lobule structures become clear and complete via the administration of meloxicam.

The hepatic lobule structures of the rat livers in the control group were clear and complete. Although the liver cells were arranged and regularly structured, the liver cells in the Al-overload group showed significant vacuolar degeneration, granular degeneration and spotty necrosis. Meloxicam had an obvious protective effect on hepatocyte injury caused by Al overload (Figure 1).

### Changes of liver function in rats

**Figure 2:**
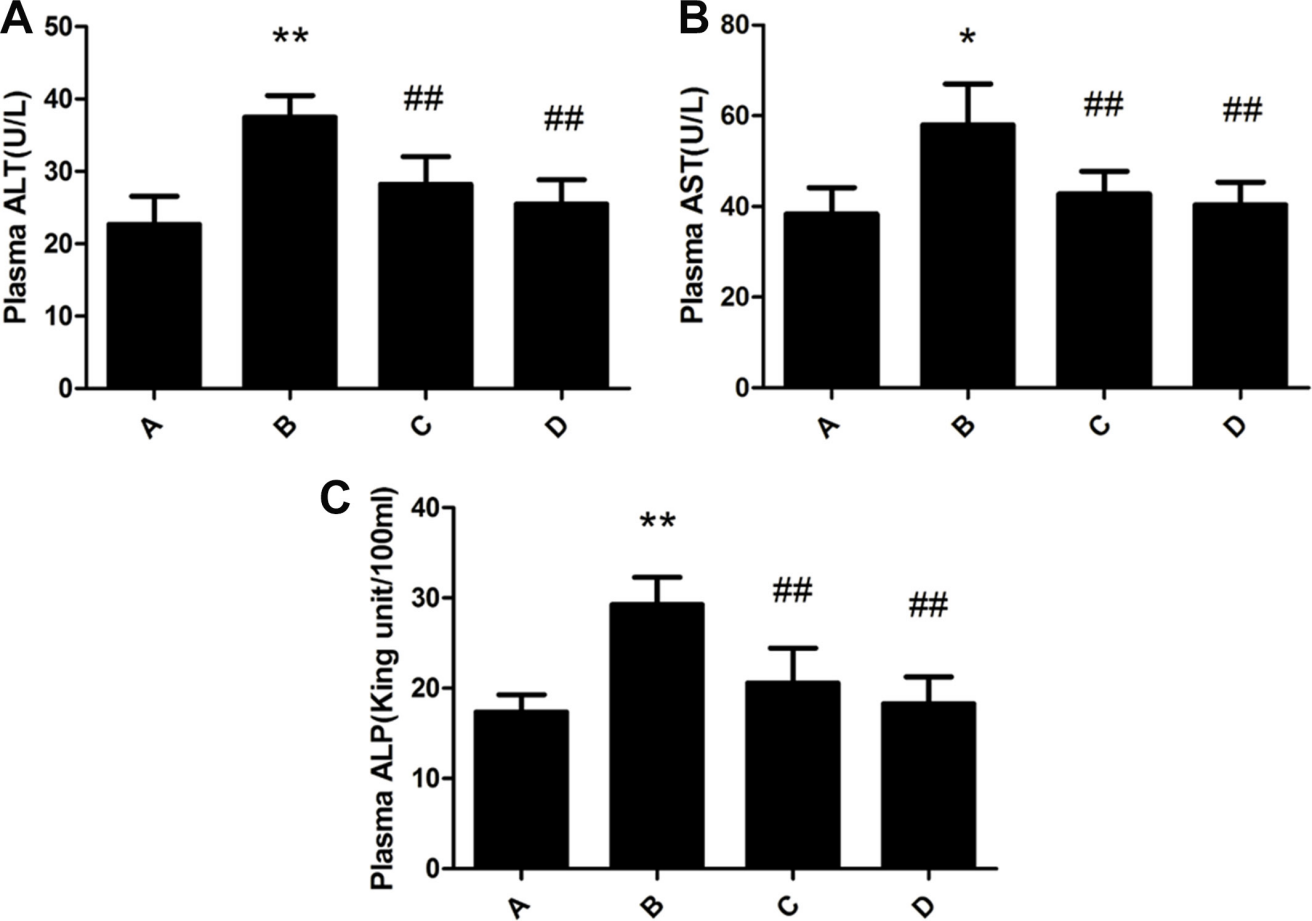
Effect of meloxicam on changes of plasma ALT, AST and ALP in chronic Al-overload rats (A) Control group; (B) Model group; (C) Al + Meloxicam 0.3 mg.kg^−1^ group; (D) Al + Meloxicam 1 mg.kg^−1^ group. (**A**) The change of ALT. (**B**) The change of AST. (**C**) The change of ALP. Data are expressed as the mean ± SD for six individual rats in each group. Compared with that of control group, the level of ALT, AST, and ALP significantly increased in model group. Compared with that of model group, the administration of meloxicam significantly decreased the levels of ALT, AST, and ALP. *P < 0.05 and ^*^P < 0.01 compared with control group, respectively. ^#^*P* < 0.05 and ^##^*P* < 0.01 compared with model group, respectively.

Blood plasma ALT, AST and ALP levels of rats in the model group were significantly increased compared with that in the control group. Administration of meloxicam significantly blunted the increase of ALT, AST and ALP levels caused by Al overload (Figure 2).

### Changes of PGE2 and cAMP contents in rat liver

**Figure 3:**
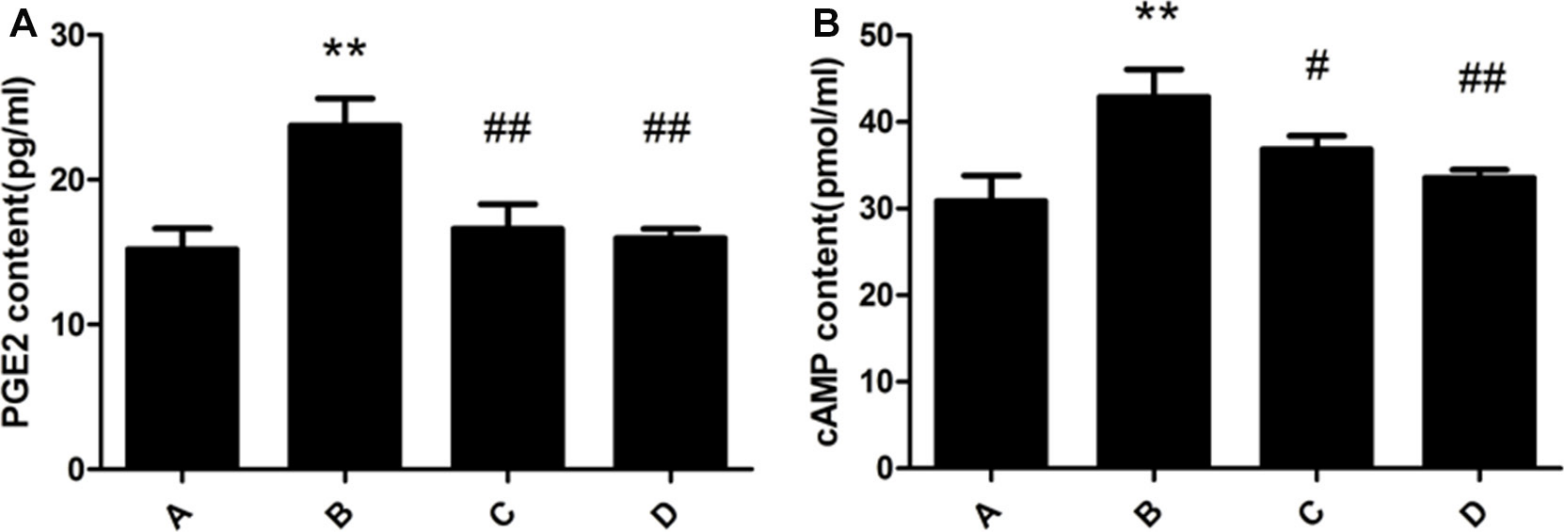
Effect of meloxicam on changes of liver PGE_2_ and cAMP in chronic Al-overload rats (A) Control group; (B) Model group; (C) Al + Meloxicam 0.3 mg.kg^−1^ group; (D) Al + Meloxicam 1 mg.kg^−1^ group. (**A**) The change of PGE2 content. (**B**) The change of cAMP content. Data are expressed as the mean ± SD for six individual rats in each group. Compared with that of control group, the contents of PGE_2_ and cAMP significantly increased in model group. Compared with that of model group, the administration of meloxicam significantly decreased the contents of PGE_2_ and cAMP. ^*^P < 0.01 compared with control group. ^#^*P* < 0.05 and ^##^*P* < 0.01 compared with model group, respectively.

PGE2 and cAMP content of rat liver in the model group were significantly increased compared with the control group. The administration of meloxicam significantly blunted the increase in PGE2 and cAMP content (Figure 3).

### Changes of SOD activity and MDA contents in rat liver

**Figure 4:**
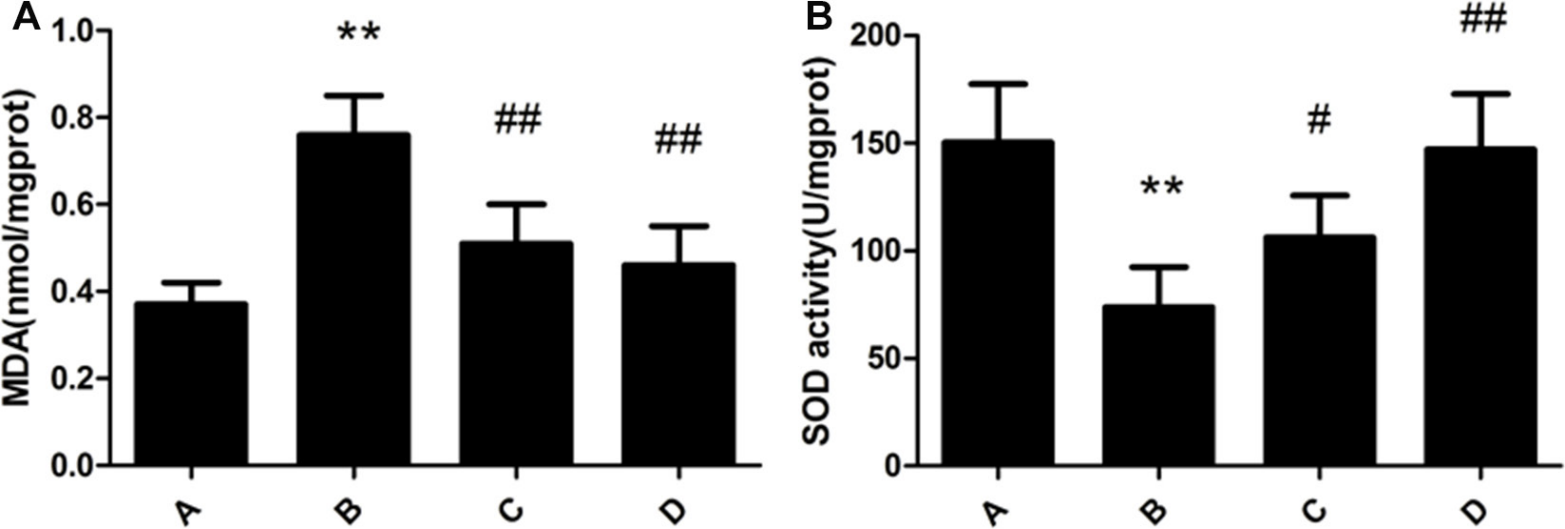
Effect of meloxicam on changes of liver MDA content and SOD activity in chronic Al-overload rats (A) Control group; (B) Model group; (C) Al + Meloxicam 0.3 mg.kg^−1^ group; (D) Al + Meloxicam 1 mg.kg^−1^ group. (**A**) The change of MDA content. (**B**) The change of SOD activity. Data are expressed as the mean ± SD for six individual rats in each group. Compared with that of control group, the content of MDA significantly increased and the activity of SOD significantly decreased in model group. Compared with that of model group, the administration of meloxicam significantly decreased the content of MDA and increased the activity of SOD. ^*^P < 0.01 compared with control group. ^#^*P* < 0.05 and ^##^*P* < 0.01 compared with model group, respectively.

SOD activity of rat liver in the model group was significantly decreased compared with the control group. MDA content of rat liver in the model group was significantly increased compared with that in the control group. Meloxicam significantly blunted the decrease of SOD activity and the increase of MDA content caused by Al overload in dose-dependent manner (Figure 4).

### Changes of TNF-α, IL-1β and IL-6 contents in rat liver

**Figure 5:**
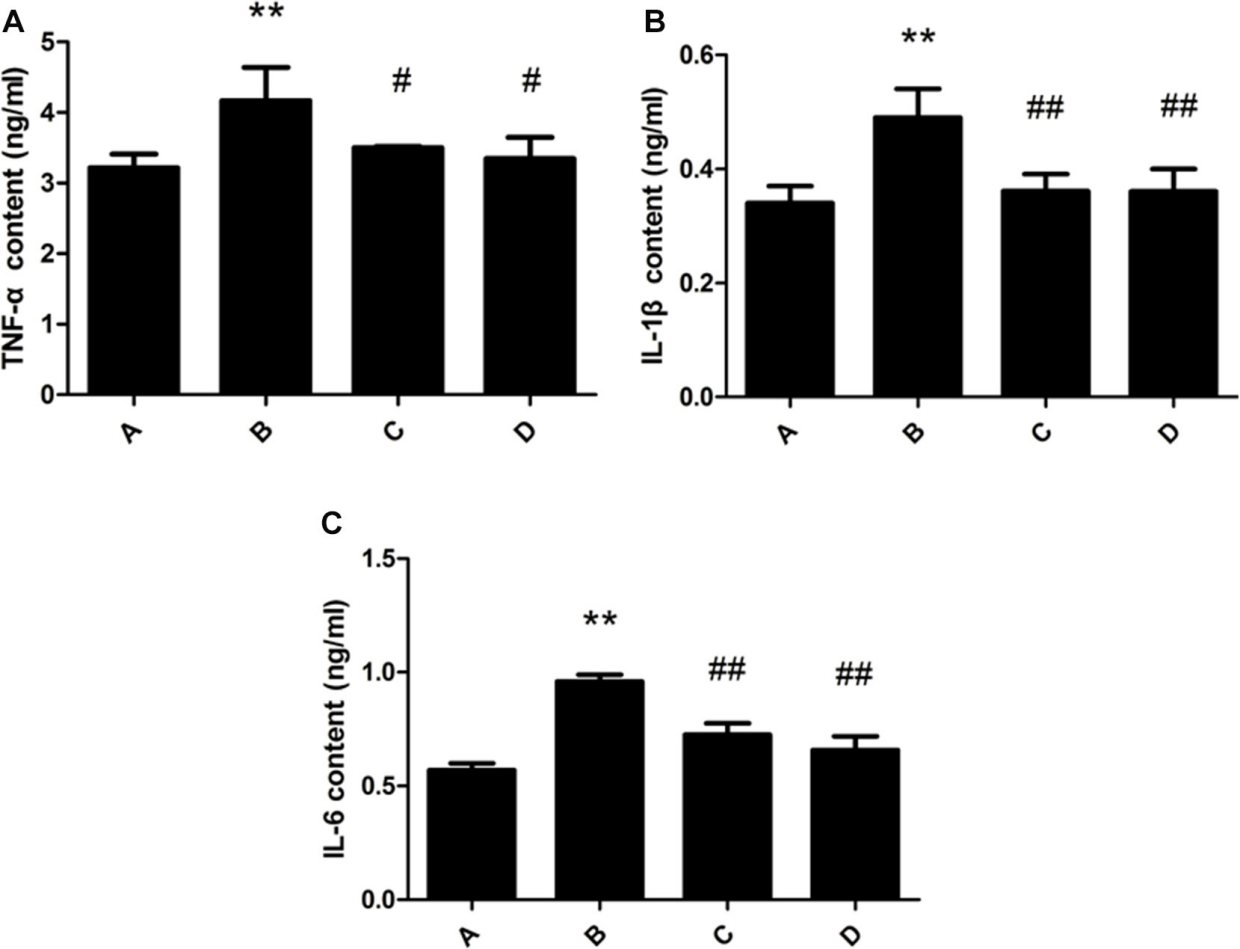
Effect of meloxicam on changes of liver TNF-α, IL-1β and IL-6 content in chronic Al-overload rats (A) Control group; (B) Model group; (C) Al + Meloxicam 0.3 mg.kg^−1^ group; (D) Al + Meloxicam 1 mg.kg^−1^ group. (**A**) The change of TNF-α content. (**B**) The change of IL-1β content. (**C**) The change of IL-6 content. Data are expressed as the mean ± SD for six individual rats in each group. Compared with that of control group, the contents of TNF-α, IL-1β and IL-6 significantly increased in model group. Compared with that of model group, the administration of meloxicam significantly decreased the contents of TNF-α, IL-1β and IL-6. ^*^P < 0.01 compared with control group. ^#^*P* < 0.05 and ^##^*P* < 0.01 compared with model group, respectively.

TNF-α, IL-1β and IL-6 content of rat liver in the model group were significantly increased compared with the control group. The administration of meloxicam, significantly blunted the increase of TNF-α, IL-1β and IL-6 content (Figure 5).

### Changes of COX2 expression in rat liver

**Figure 6:**
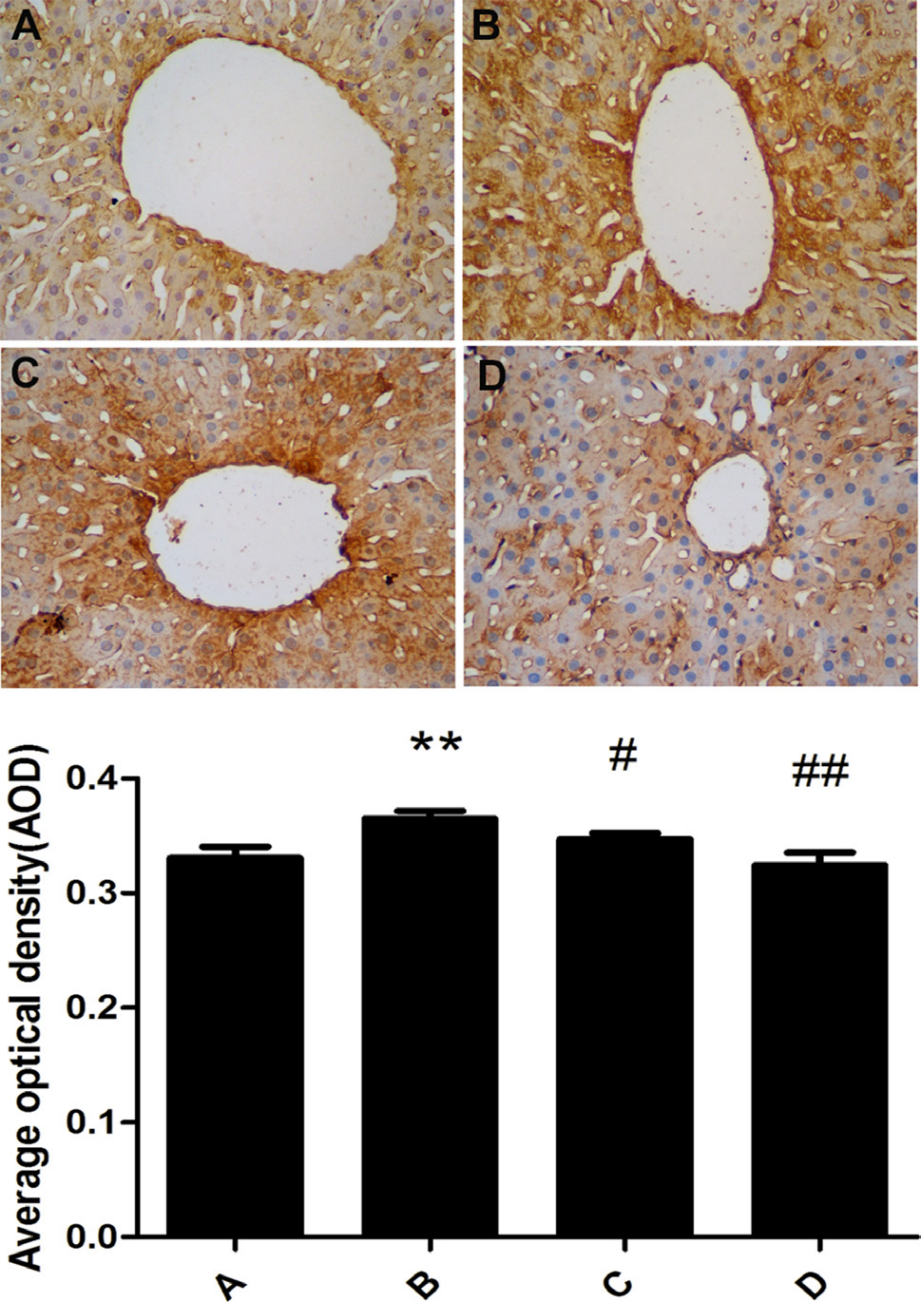
Effect of meloxicam on changes of liver COX2 protein expression in chronic Al-overload rats (A) Control group; (B) Model group; (C) Al + Meloxicam 0.3 mg.kg^−1^ group; (D) Al + Meloxicam 1 mg.kg^−1^ group. Change of COX2 protein expression was measured by immunohistochemistry (400×), the AOD of COX2 were analyzed by IPP. Data are expressed as mean ± SD of three individual experiments. Compared with that of control group, COX2 protein expression significantly increased in model group. Compared with that of model group, the administration of meloxicam significantly decreased COX2 protein expression. ^*^P < 0.01 compared with control group. ^#^*P* < 0.05 and ^##^*P* < 0.01 compared with model group, respectively.

COX2 protein was mainly expressed in the cytoplasm of rat liver cell. The expression of COX2 in the model group was significantly increased compared with the control group. The administration of meloxicam significantly blunted the increase of COX2 protein expression caused by Al overload (Figure 6).

### Changes of EP_1,2,3,4_ expression in rat liver

**Figure 7:**
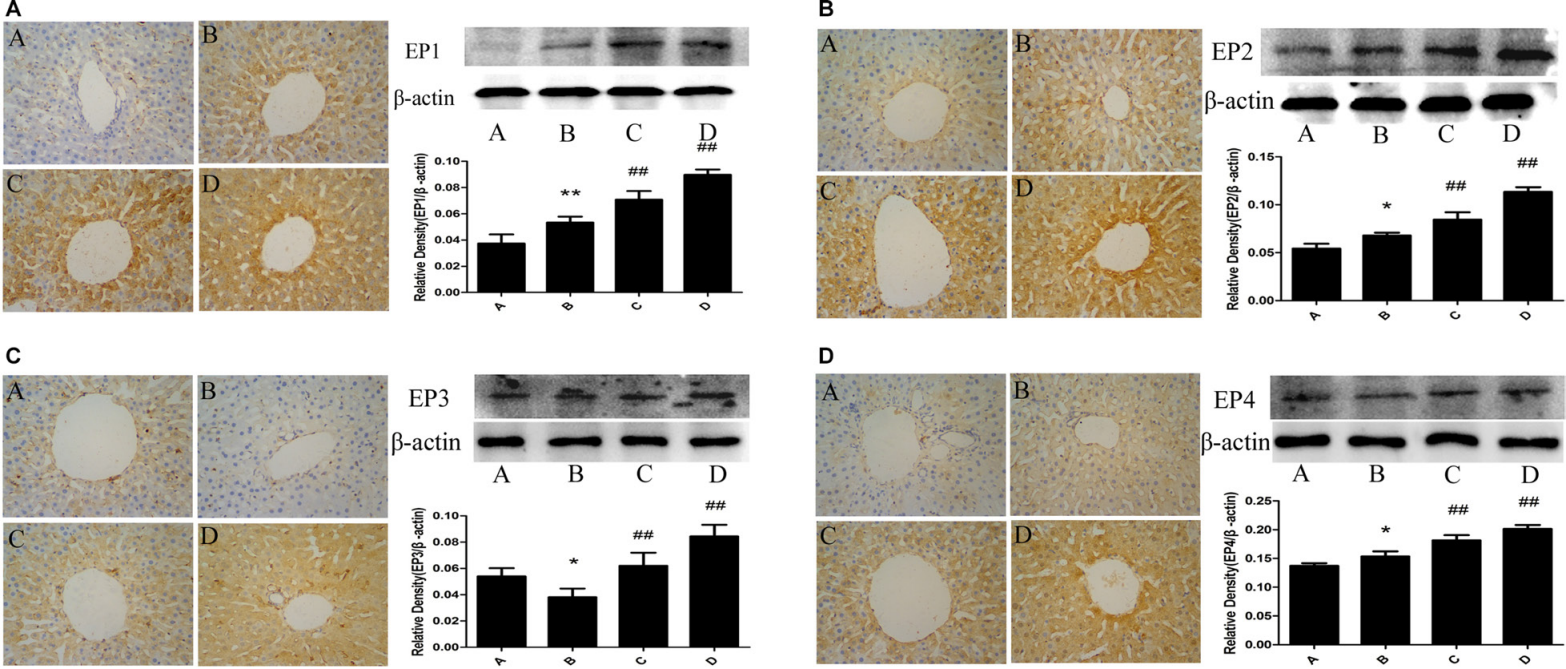
Effect of meloxicam on changes of liver EP_1,2,3,4_ protein expression in chronic Al-overload rats (A) Control group; (B) Model group; (C) Al + Meloxicam 0.3 mg.kg^−1^ group; (D) Al + Meloxicam 1 mg.kg^−1^ group. Change of EP_1_(a), EP_2_(b), EP_3_(c) and EP_4_(d) protein expressions were measured by immunohistochemistry (400×) and WB. The relative protein level of EP_1,2,3,4_ were standardized to endogenous β-actin protein for each sample. Data are expressed as mean ± SD of three individual experiments. Compared with that of control group, EP_1,2,4_ protein expression significantly increased and EP_3_ significantly decreased in model group. Compared with that of model group, the administration of meloxicam significantly increased EP_1,2,3,4_ protein expression. *P < 0.05 and ^*^P < 0.01 compared with control group, respectively. ^#^*P* < 0.05 and ^##^*P* < 0.01 compared with model group, respectively.

There was a little expression of EP_1,2,3,4_ of rat liver in the control group. EP_1,2,4_ expression of rat liver in the model group was significantly increased and EP_3_ expression of rat liver in the model group was significantly decreased compared with the control group. The administration of meloxicam significantly increased the expression of EP_1,2,3,4_ compared with the model group (Figure 7).

### Changes of PKA expression in rat liver

**Figure 8:**
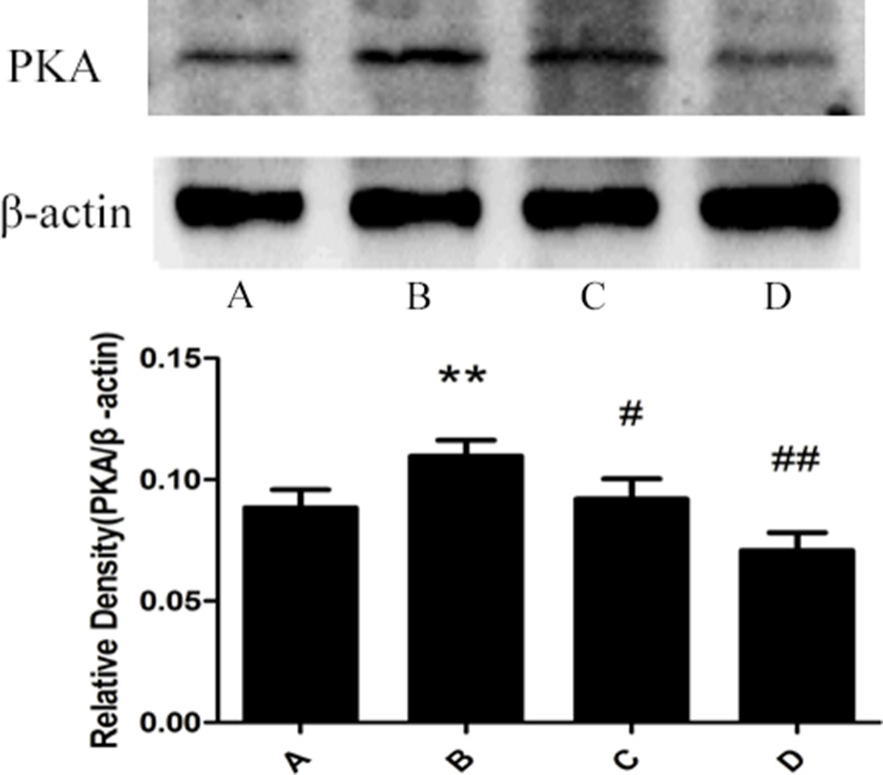
Effect of meloxicam on changes of liver PKA protein expression in chronic Al-overload rats (A) Control group; (B) Model group; (C) Al + Meloxicam 0.3 mg.kg^−1^ group; (D) Al + Meloxicam 1 mg.kg^−1^ group. The protein expression of PKA was measured by Western blot. The relative protein level of PKA was standardized to endogenous β-actin protein for each sample. Data are expressed as mean ± SD of three individual experiments. The expression of PKA in the model group was significantly increased. The administration of meloxicam significantly blunted the increase of PKA expression. ^*^P < 0.01 compared with control group. ^#^*P* < 0.05 and ^##^*P* < 0.01 compared with model group, respectively.

The result from the western blotting detection indicated that there is little expression of PKA of rat liver in the control group. The expression of PKA in the model group was significantly increased compared with the control group. The administration of meloxicam significantly blunted the increase of PKA expression caused by Al (Figure 8).

## DISCUSSION

Several studies showed similar results that Al overload could cause liver damage [6–9]. Our experiment results also showed obvious vacuolar degeneration, granular degeneration and spotty necrosis in chronic Al-overload rat hepatocytes and the levels of ALT, AST and ALP in plasma were significantly increased in Al-treated rat. These results further confirmed that chronic Al overload could cause liver damage, so the liver function protection should be paid more attention for professional worker subjected to long-term Al exposure [5].

The mechanism of Al producing toxic reaction might be attributed to produce more reactive oxygen species (ROS). Superoxide dismutase (SOD), one type of the antioxidant enzyme, is capable of detoxifying superoxide. Malondialdehyde (MDA) is a product of enzyme reactions which reflects extensive peroxidations [22]. Some studies have shown that Al induced toxicity is mediated by free radical generation and alterations in antioxidant enzymes [23–24]. The conformation of the SOD molecule is altered by the formation of the Al-SOD complex, which might decrease the activity of the SOD [25]. Al easily occupies the binding sites of iron, as a result, the concentration of free Fe^2+^ is significantly increased to produce more ROS to cause cell injury [26–27]. Our previous study and many other studies have shown that Al may disrupt the homeostasis of metal leads to oxidative stress cause liver damage [13, 28–29]. In the present study, we also found that the activity of SOD was significantly decreased and the content of MDA was significantly increased in Al-overload rats. Those results further indicated that oxidative stress may be involved in chronic liver injury caused by Al. Additionally, oxidative stress also can promote inflammation. Metal ion transporters, such as the divalent metal-ion transporter-1 (DMT-1), it could be activated by inflammation to increase metal ions into the liver to cause oxidative stress [30]. Therefore, oxidative stress and inflammation formed a vicious circle.

Inflammation is also a very important reason involved in the process of liver injury. Some studies have found that Al is mainly accumulated in macrophages [31]. Macrophages are the main source of COX2 in liver [32]. PGs, ROS, cytokines and various kinds of protease were produced in macrophages to release to effect on all cells [33]. Al could decrease the SOD activity to produce more ROS to increase the expression of COX2 in macrophages. So, the activation of macrophages may be the important reason of inflammation in chronic Al-overload rat liver.

ROS could be used as the second messenger activating NF-κB to increase the transcription of genes [34]. There are two NF-κB binding sites in the COX2 promoter, and NF-κB combined with the sites could increase the transcription of COX2 [35]. PGE2 is one of the most abundant and active of the PGs in COX2 downstream [36–37]. PGE2 is also involved in various pathophysiological processes, include inflammation and oxidative stress [38–41]. PGE2 has been found to participate in a variety of liver damage processes [19–21]. PGE2 receptors are designated as EP_1_, EP_2_, EP_3_ and EP_4_ [42–43]. In the present study, we found that the expression of COX2, protein kinase A(PKA) and EP_1,2,4_ were significantly increased, the expression of EP_3_ were significantly decreased, and the content of PGE2 and cyclic adenosine monophosphate (cAMP) were significantly increased in Al-overload rats liver. EP_1_ could increase the intracellular content of Ca^2+^. The excessive Ca^2+^ could injury mitochondria to produce more ROS to damage the liver cells [44–45]. The EP_2_,_4_ can stimulate adenylate cyclase(AC) with a subsequent increase of intracellular cAMP which increases PKA. p38 MAPK is involved in the activation of apoptosis because of being activated by cAMP/PKA signaling pathway, and it activates caspase-1, caspase-3 and caspase-11 and causes the produce of TNF-α, IL-1 and IL-6 in parenchymal cells [46]. p38 MAPK is also activated in macrophages to produce more inflammatory cytokines, such as TNF-α, IL-1, IL-6, and others [47]. However, EP_3_ could reduce the content of cAMP and PKA. The decrease of cAMP and PKA might inhibit the activation of cAMP/PKA signaling pathway. These previous results suggested that EP_1,2,4_ might promote liver injury and EP_3_ might improve liver injury. In our study, we also found that the content of TNF-α, IL-1β and IL-6 were significantly increased in Al-overload rats. Considering that inflammatory cytokines had a stimulating effect on the accumulation of neutrophils to aggravate the damage of inflammation in the liver [48], our results indicate that activation of the PGE2-EP_1,2,4_-cAMP/PKA signaling pathway can cause serious liver injury by stimulation of inflammation and oxidative stress by Al.

Our experimental results showed that the treatment of meloxicam significantly alleviated vacuolar degeneration, granular degeneration and spotty necrosis of rat hepatocyte and decreased the levels of ALT, AST and ALP in Al-treated rats plasma, and it also significantly blunted the decrease of SOD activity and the increase of MDA, TNF-α, IL-1β and IL-6 content in Al-treated rats liver. Moreover, our experimental results also found that meloxicam could significantly decrease the expression of COX2 and PKA, blunt the increase of PGE2 and cAMP, and increase the EP_1,2,3,4_ expression.

Chronic Al overload could cause the decrease of EP_3_ expression by the desensitization of EP_3_ receptor (down-regulation of EP_3_) long-term exposure to high PGE2 level [49]. This is the normal regulation of receptor [50]. However, the increase of EP_1,2,4_ expressions in aluminium overload rats suggest that there is a dysregulation of EP_1,2,4_ expressions long-term exposure to high PGE2 level. But the reason about the dysregulation of EP_1,2,4_ expressions is not clear. Some studies also have proved that EP_1,2,4_ would promote inflammation and EP_3_ would inhibit inflammation [51–53]. Together with our studies, EP_1,2,4_ expressions could promote liver injury and EP_3_ expression could protect liver against injury caused by chronic Al overload through their own signaling pathway. Meloxicam treatment inhibited the COX2 activity and decreased the PGE2 level. The decrease of PGE2 level caused the up-regulation of EP_1,2,3,4_. Perhaps, the effects of the increase of protective EP_3_ expression exceed the effects of the increase of injury EP_1,2,4_ expressions. Finally, the cAMP/PKA pathway is inhibited. As a result, the meloxicam protected the liver against the injury caused by Al overload. Moreover, some researchers also have indicated that EP_3_ expression might inhibit the proinflammatory effect of EP_2,4_ [54]. These results together indicated that meloxicam can protect liver injury against Al exposure *via* inhibiting the COX2 to reduce PGE2 and increase the expression of EP_3_ to alleviate inflammation and oxidative stress through reconstructing the balance of COX2-PGE2-EPs-cAMP/PKA signaling pathway.

In summary, the present results suggested that meloxicam has an obvious protective effect on rat liver damage caused by Al. The protective mechanism of meloxicam on liver injury is attributed to reconstruct the balance of COX2-PGE2-EPs-cAMP/PKA signaling pathway and further to reduce inflammation and oxidative stress in chronic Al-overload rat. These findings pointed out that the COX2-PGE2-EPs-cAMP/PKA signaling pathway which is associated with the inflammation and oxidative stress is a potential therapeutic target for chronic non-infection liver diseases.

## MATERIALS AND METHODS

### Animals

Rats were housed in the barrier housing facility, and it has in keeping with national standard “Laboratory Animal-Requirements of Environment and Housing Facilities” (GB 14925–2001). The care of laboratory animal and the animal experimental operation have conforming to “Chongqing Administration Rule of Laboratory Animal”. The experimental procedures were approved by the animal laboratory administrative center and the institutional ethics committee of Chongqing Medical University (License number: SYXK YU 2010–0001) and also in accordance with the National Institutes of Health guidelines.

Twenty-four male adult SD rats (*obtained from the animal laboratory centre of Chongqing Medical University*), weighing 220–250 g, were randomly and equally divided into the following 4 groups: control group, model group, M-0.3 group (Al+Meloxicam 0.3 mg·kg^−1^ group), and M-1 group (Al+Meloxicam 1 mg·kg^−1^ group), *n* = 6 for each groups.

### Chemical

AlCl_3_·6H_2_O (*Sinopharm Chemical Reagent Co., Ltd., China*) and sodium gluconate (*Beijing Qing Sheng Da Chemical Technology Co., Ltd., China*) were of analytical grade. Meloxicam was purchased from Kunshan Rotam Reddy Pharmaceutical Co., Ltd, China. According to previously reported methods [13, 17], an aluminium gluconate solution (20 mg Al^3+^·ml^−1^) was prepared on the day of the experiments by adding 17.9 g of AlCl_3_· 6H_2_O and 9.9 g of sodium gluconate to 100 ml of double distilled water (ddH_2_O), and the pH of the solution was adjusted to approximately 6.0.

### Establishment of animal models

After 3 days of acclimatisation, the rats were treated intragastrically once a day, 5 d per week for 20 weeks with the following solutions: the model group received 10 ml·kg^−1^ aluminium gluconate solution (200 mg Al^3+^·kg^−1^), and the control group received the same volume of a sodium gluconate solution. The M-0.3 group and M-1 group were treated intragastrically with 0.3 and 1 mg.kg^−1^ meloxicam, respectively, 30 min after intragastric administration of aluminium gluconate (200 mg Al^3 +^·kg^−1^).

### Liver function tests

On the second day after the cessation of Al gluconate administration, 6 rats from each group were chosen, and 1 ml blood was collected from each rat orbit. The blood plasma levels of alanine aminotransferase (ALT), aspartate aminotransferase (AST) and alkaline phosphatase (ALP) were measured according to a kit manual (*Jiancheng Bioengineering Ltd, Nanjing, China*).

### Histopathological observation

On the second day after the cessation of Al gluconate or sodium gluconate administration, 3 rats from each group were chosen for histopathological observation. The rats were intraperitoneally anesthetised with 4% chloral hydrate (10 ml·kg^−1^) and transcardially perfused with heparinised saline (100 ml) followed by 4% paraformaldehyde in phosphate-buffered saline (200 ml). The rat livers were removed and stored in the same fixative solution. The liver tissue was sliced into 5-μm-thick sections for haematoxylin and eosin staining (H.E). Hepatic pathological changes were observed under light microscopy.

Before perfusion, a portion of the fresh liver was taken to prepare for subsequent experiments including measure of MDA content and SOD activity and the test of WB and ELISA.

### Immunohistochemical staining test

Immunohistochemistry was performed to investigate the expression of COX2 and EP_1,2,3,4_ in the rat livers. Briefly, liver sections of 3 rats from each group were dewaxed and rehydrated in decreasing concentrations ethanol. Then the sections were blocked for endogenous peroxidase in 3% H_2_O_2_ in methanol for 20 min at room temperature. Slides were washed with PBS for three times and pre-incubated in 1% serum for 30 min at room temperature. Thereafter, the sections were incubated with primary antibodies COX2 (dilution 1:50, *Santa, USA*) and EP_1,2,3,4_ (dilution 1:100, *Santa, USA*) overnight at 4°C. Then, the sections were incubated with biotinylated secondary antibody (dilution 1:100) for 30 min at 37°C, and incubated with streptavidin for 20 min, and then rinsed for another 3 min × 3 with PBS before reaction with DAB solution. The sections were counterstained with hematoxylin and then observed under a microscope. The average optical density (AOD) was used to represent the expression of COX2 in the sections. The AOD of COX2 in the sections were analyzed by Image Pro Plus6.0 (IPP).

### Measurement of malondialdehyde (MDA) content and superoxide dismutase (SOD) activity

Rat livers from each group of were removed on the second day after Al gluconate administration was completed (*n* = 6). The SOD activity and MDA content were detected according to the instruction manual of a kit (*Jiancheng Bioengineering Ltd, Nanjing, China*). The protein content was measured using a BCA protein assay kit *(Beyotime, China)*.

### Enzyme-linked immunosorbent assay (ELISA)

Rat livers from each group of rats (*n* = 6)were removed on the second day after Al gluconate administration was completed. PGE2 (*TaKaRa Japan*), cAMP (*R&D Systems China shanghai, China*), TNF-α (*HuaMei Bioengineering Ltd, Wuhan, China*), IL-1β (*HuaMei Bioengineering Ltd, Wuhan, China*) and IL-6 (*HuaMei Bioengineering Ltd, Wuhan, China*) were detected with ELISA kits.

### Western blotting test

Fifty mg of rat liver (*n* = 3) were added to 0.5 ml of tissue lysate solution for protein extraction and centrifugation at 12,000 × g for 10 min at 4°C, and the supernatant was used for detection of protein concentrations with a BCA protein assay kit *(Beyotime, China)*. A 10 μL sample of protein was separated by sodium dodecyl sulphate polyacrylamide gel electrophoresis (SDS-PAGE) and transferred to PVDF membranes (*Millipore, USA*). The membranes were blocked with 5% BSA for 1 h at room temperature and then probed with specific primary antibodies, including anti-EP_1_, EP_2_, EP_3_, EP_4_ and PKA (1:300; *Santa, USA*) and β-actin (1:3000; *Proteintech, USA*) overnight at 4°C. The membranes were washed three times in TBST and incubated with HRP-conjugated secondary antibodies at room temperature for one hour. Following four washes in TBST, protein signals were visualized by ECL *(Bio-Rad, USA)*.

### Statistical analysis

The results were expressed as the means ± standard deviation (SD) and were analysed with SPSS 12.0 (SPSS Inc. Chicago, US). Within-group variances were compared using Dennett's *t-test*. Statistical significance was represented by *P* < 0.05.
